# The global response: How cities and provinces around the globe tackled Covid-19 outbreaks in 2021

**DOI:** 10.1016/j.lansea.2022.100031

**Published:** 2022-06-22

**Authors:** Nityanand Jain, I-Chun Hung, Hitomi Kimura, Yi Lin Goh, William Jau, Khoa Le Anh Huynh, Deepkanwar Singh Panag, Ranjit Tiwari, Sakshi Prasad, Emery Manirambona, Tamilarasy Vasanthakumaran, Tan Weiling Amanda, Ho-Wei Lin, Nikhil Vig, Nguyen Thanh An, Emmanuel Uwiringiyimana, Darja Popkova, Ting-Han Lin, Minh Anh Nguyen, Shivani Jain, Tungki Pratama Umar, Mohamed Hoosen Suleman, Elnur Efendi, Chuan-Ying Kuo, Sikander Pal Singh Bansal, Sofja Kauškale, Hui-Hui Peng, Mohit Bains, Marija Rozevska, Thang Huu Tran, Meng-Shan Tsai, Pahulpreet, Suvinai Jiraboonsri, Ruo-Zhu Tai, Zeeshan Ali Khan, Dang Thanh Huy, Supitsara Kositbovornchai, Ching-Wen Chiu, Thi Hien Hau Nguyen, Hsueh-Yen Chen, Thanawat Khongyot, Kai-Yang Chen, Dinh Thi Kim Quyen, Jennifer Lam, Kadek Agus Surya Dila, Ngan Thanh Cu, My Tam Huynh Thi, Le Anh Dung, Kim Oanh Nguyen Thi, Hoai An Nguyen Thi, My Duc Thao Trieu, Yen Cao Thi, Thien Trang Pham, Koya Ariyoshi, Chris Smith, Nguyen Tien Huy

**Affiliations:** a Faculty of Medicine, Riga Stradins University, 16 Dzirciema iela, Riga LV-1007, Latvia; b Online Research Club (https://www.onlineresearchclub.org/), Nagasaki, Japan; c Department of Public Health Medicine, Faculty of Medicine, University of Tsukuba, Tsukuba, Japan; d Alice Lee Centre for Nursing Studies, Yong Loo Lin School of Medicine, National University of Singapore, Singapore; e School of Medicine, College of Medicine, Taipei Medical University, Taipei, Taiwan; f Department of Biostatistics, Virginia Commonwealth University, School of Medicine, Virginia 23224, USA; g B.P. Koirala Institute of Health Sciences, Dharan 56700, Nepal; h National Pirogov Memorial Medical University, Vinnytsya, Ukraine; i College of Medicine and Health Sciences, University of Rwanda, Kigali, Rwanda; j Global Scholars Clinical Research Training Program, Harvard Medical School, Boston, MA, USA; k Faculty of Dentistry, National Dental College and Hospital, Dera Bassi, Mohali, Punjab, India; l College of Medicine and Pharmacy, Duy Tan University, Da Nang City, Vietnam; m Hanoi Medical University/Hanoi University of Public Health, Vietnam; n Genesis Institute of Dental Sciences and Research, Firozpur, Punjab, India; o Faculty of Medicine, Sriwijaya University, Palembang, Indonesia; p Nelson R. Mandela School of Medicine, University of KwaZulu-Natal, Durban, South Africa; q University of Medicine and Pharmacy Ho Chi Minh City, Ho Chi Minh City, Vietnam; r Faculty of Medicine, Chulalongkorn University, Bangkok, Thailand; s Shadan Institute of Medical Sciences, Hyderabad, India; t International University of Health and Welfare, Chiba, Japan; u School of Pharmacy, Walailak University, Nakhon Si Thammarat, Thailand; v Faculty of Arts and Science, University of Toronto, Toronto, Canada; w Giri Emas Hospital, Singaraja City, 81171 Buleleng, Bali, Indonesia; x School of Medicine and Pharmacy, The University of Da Nang, Da Nang City 50000, Vietnam; y Hue University of Medicine and Pharmacy, Hue City, Vietnam; z Faculty of Medicine, Tay Nguyen University, Buon Ma Thuot City, Vietnam; aa School of Tropical Medicine and Global Health, Nagasaki University, Japan; bb Department of Clinical Medicine, Institute of Tropical Medicine, Nagasaki University, Nagasaki, Japan; cc Department of Clinical Research, London School of Hygiene and Tropical Medicine Faculty of Infectious and Tropical Diseases, London, United Kingdom

**Keywords:** COVID-19, Global response, Cities, Provinces, Delta variant, Testing, Management, Swiss Cheese, Transmission

## Abstract

**Background:**

Tackling the spread of COVID-19 remains a crucial part of ending the pandemic. Its highly contagious nature and constant evolution coupled with a relative lack of immunity make the virus difficult to control. For this, various strategies have been proposed and adopted including limiting contact, social isolation, vaccination, contact tracing, etc. However, given the heterogeneity in the enforcement of these strategies and constant fluctuations in the strictness levels of these strategies, it becomes challenging to assess the true impact of these strategies in controlling the spread of COVID-19.

**Methods:**

In the present study, we evaluated various transmission control measures that were imposed in 10 global urban cities and provinces in 2021– Bangkok, Gauteng, Ho Chi Minh City, Jakarta, London, Manila City, New Delhi, New York City, Singapore, and Tokyo.

**Findings:**

Based on our analysis, we herein propose the population-level Swiss cheese model for the failures and pitfalls in various strategies that each of these cities and provinces had. Furthermore, whilst all the evaluated cities and provinces took a different personalized approach to managing the pandemic, what remained common was dynamic enforcement and monitoring of breaches of each barrier of protection. The measures taken to reinforce the barriers were adjusted continuously based on the evolving epidemiological situation.

**Interpretation:**

How an individual city or province handled the pandemic profoundly affected and determined how the entire country handled the pandemic since the chain of transmission needs to be broken at the very grassroot level to achieve nationwide control.

**Funding:**

The present study did not receive any external funding.

**Research in context**

**Evidence before this study**

Multiple studies previously have reported the importance of appropriate implementation and enforcement of public health measures to tackle the spread of COVID-19 in communities. However, these studies have either compared and evaluated the efficiency of these measures at the country level or looked at individual cities. Additionally, in many countries, there were major differences in the adaptation of the guidelines amongst different regions/cities which evolved rapidly with the changing epidemiological situation. Hence, it is important to evaluate and compare the effectiveness of the responses that were adopted at the city/province level which represents the lowest level at which the chain of viral transmission needs to be broken. We assessed the COVID-19 measures that were implemented in eight global metropolitan cities and provinces where major outbreaks were reported (>5000 confirmed cases daily) along with two metropolitans with a controlled outbreak. These urban centers were chosen due to the effective mass communication and healthcare measures that they have in place. The data presented here correlated mostly with the management of the delta variant of COVID-19 since we analyzed measures from January to November 2021. We analyzed the eight most impactful public health measures (with sub-criteria) in all these ten cities and provinces and correlated them with the outcome and impact on COVID-19 management.

**Added value of this study**

To our knowledge, this is the first time such a descriptive comparative analysis has been reported at the city/province level as we show the differences in the management approach and their impact on controlling the spread of the virus. We found that whilst all the evaluated cities/provinces took a personalized approach to manage the pandemic, certain measures like contact tracing and vaccination promotion proved to be more impactful to break the transmission chain than others like mass testing. Furthermore, the measures taken to reinforce the barriers were adjusted continuously based on the evolving epidemiological situation. Based on these findings, we herein propose the population-level Swiss Cheese model for breaking the chain of transmission at the grass-root level, which eventually influences the effectiveness of the nationwide control.

**Implications of all the available evidence**

Dynamic enforcement and monitoring of breaches of each barrier of protection are critical in ensuring effective management of major outbreaks in cities/provinces. The impact of breaches in one intervention/layer would be minimized by the next intervention only if the breach doesn't overlap each other, in which case, there would be a higher likelihood of Covid-19 transmission. The staggering increase in daily case counts in different cities is often the result of a combination of failures in different interventions and seldom due to a single enforcement cause. Future research should focus on adjusting previously proposed transmission models to a city environment.

Alt-text: Unlabelled box

## Introduction

A highly contagious and infectious virus, the Severe Acute Respiratory Syndrome Coronavirus-2 (SARS-CoV-2) was first detected in December of 2019 in Wuhan, China when multiple clusters of viral pneumonia were reported with an unknown etiological agent.^1^^,^^2^ Belonging to the *Coronaviridae* family, SARS-CoV-2 is a beta coronavirus with an exceptionally huge positive-sense single strand of the RNA genome, which is encased in an envelope that has a rim of projections resembling the solar corona.^3^^,^^4^ The beta coronaviruses are primarily zoonotic viruses that can be transmitted from animals to humans due to a spill over event, thereby causing human disease.^5^ Due to the errors in its RNA structure, the virus is prone to undergoing rapid mutations, leading to the emergence of highly contagious and transmissible variants.^3^^,^^6^^,^^7^

SARS-CoV-2 caused COVID-19 disease is characterized by a febrile infection mainly associated with respiratory symptoms, although it can trigger an excessive immune response (cytokine storm), leading to multiple organ failure and subsequent death.^8^^,^^9^ The high rate of human-to-human transmission, relative lack of immunity against the virus, asymptomatic infections, and the fact that people can be infectious even prior to symptom onset make the SARS-CoV-2 even more concerning and difficult to control.^10^ Initial early strategies to control the spread of the virus in most countries included a mix of international travel restrictions, quarantine imposition on people with a history of travel to countries with high viral prevalence, and contact tracing.^11^ However, as the outbreak spread and progressed into the local communities (mainly due to slow imposition of restrictions, non-adherence to rules, and relaxed contact tracing), diagnostic testing was increased and prioritized especially for people developing respiratory symptoms like cold, cough, and/or fever. This led to discrete acceleration events which were managed with stricter regulations including lockdowns, use of face masks and disinfectants, switching work and education to online mode, and social distancing.^12^^,^^13^

This strategy of “limiting contact” enabled the authorities to reduce the frequency and duration of contacts, thereby reducing the number of nodes for viral replication i.e., reducing the basic viral reproduction number (R_0_).^13^ However, given the heterogeneity in the frequency, duration, and timing of the lockdown across the globe, it is difficult to quantify the effects of the lockdown on the control of COVID-19 infection.^13^ Furthermore, as the number of new daily cases detected and the number of new daily hospitalizations fluctuated, the strictness levels in the lockdowns were also altered to suit the emerging situation. The lockdowns additionally, resulted in many people losing their livelihood whilst taking a heavy toll on an individual's mental well-being, making the benefit-to-risk analysis of the limiting contact strategy extremely difficult to assess.

By the end of 2020, multiple vaccines were approved by the World Health Organization (WHO) on an emergency use basis,^14^ which provided governments with an alternative strategy to ease the burden on the already stretched healthcare system. However, inequality in vaccine distribution^15^ and misinformation-led vaccine hesitancy^16^ only added further complexity to achieving herd immunity.^17^ All these variables allowed the virus to infect a larger proportion of the population in a relatively shorter period, thereby, increasing the mutation densities of the virus,^18^ and the emergence of wave peaks of the disease in countries across the globe. These peaks pressurized the medical community and hospitals leading to a shortage of staff, oxygen, and other essential medical supplies.^19^^,^^20^ Therefore, to prevent and extinguish the next waves, it becomes essential to strictly control the number of cases within a country/region combined with the effective tracing of newly infected imported cases.^21^ However, not all cities and countries have been able to effectively follow or implement this strategy.

Epidemiological modeling has demonstrated that the inter-wave strolling period is crucial in determining the dynamics of the subsequent waves, with the model being strongly dependent on the testing strategy and quantification.^21^ Furthermore, the viral spread control and testing strategy during this strolling period are far more critical in avoiding the next wave than ramping up during the wave period since the latter can only aid in containing the wave and not prevent it.^21^ Previous studies during the early stages of the pandemic have shown the utility of qualitative, comparative reviews in influencing the policy-making decisions and controlling the spread of the pandemic.^22^^,^^23^

Hence, in the present study, we investigated and compared the control measures and the testing strategies that were adopted by 10 urban metropolitan cities/provinces from across the globe during COVID-19 outbreaks in 2021 (mostly delta variant dominated). Such comparative epidemiological studies, in our view, can support the global efforts to suppress the future waves and help the policymakers in preventing the next one by understanding the differences and effectiveness of different control and testing models that were adopted by each of these respective global cities/provinces.

## Methods

### Selection criteria of the cities/provinces

In the present study, cities/provinces were selected using convenience sampling that relied on (a) cities fulfilling the inclusion criteria based on the peak reported number of daily COVID-19 confirmed cases during the defined wave period; (b) free and public availability of reliable data concerning the parameters studied; (c) inclusion of only urban centers due to the presence of well-established mass communication and healthcare infrastructure; and (d) collaboration with a local collaborator(s) from the respective city/province for collection and subsequent verification of the data.

Based on this, eight cities/provinces were selected that had suffered from major COVID-19 outbreaks in 2021 with peak daily new confirmed cases being higher than 5000 cases. Additionally, two cities with a relatively controlled outbreak (Singapore, Singapore and Manila, Philippines) were included to study and compare the differences in the testing and control strategies amongst the different cities and provinces. All the data presented in the study pertains to the data from the outbreaks reported in 2021.

### Wave definition and data curation

The COVID-19 outbreak timeline was defined by visually analyzing the trend chart of different cities/provinces from official government sources/dashboards for as long as the daily new confirmed cases remained more than 1000 cases. The data for confirmed daily cases were obtained from respective government sources (Table 1). The data presented correlated mostly with the management of the delta variant of COVID-19 since the Omicron variant was first reported to WHO on 24th November 2021, followed by its subsequent declaration as a Variant of Concern (VOC) on 26th November 2021.^24^ This makes the exact borderline for the adoption of omicron-specific control measures difficult to estimate. To remove this ambiguity, we investigated only the data available before November 2021 i.e., from 1st January 2021 to 31st October 2021. All the collected data along with sources was stored in an online Google Drive Word document. The graphs were prepared using MS Excel (Microsoft 365 for Windows 10, Microsoft Corp., USA).

**Table 1:** Characteristics of investigated cities/provinces and the source of confirmed daily cases.

Sr. No.	City/Provinces	Country	Type	Population^a^	Data source for daily confirmed COVID-19 cases
1.	Bangkok	Thailand (Asia)	Major Outbreak	5.5 million	https://covid19.ddc.moph.go.th/api/Cases/timeline-cases-by-provinces
2.	Gauteng	South Africa (Africa)	Major Outbreak	15.5 million	https://github.com/dsfsi/covid19za/blob/master/data/district_data/provincial_gp_cumulative.csv
3.	Ho Chi Minh City	Vietnam (Asia)	Major Outbreak	3.5 million	https://ncov.vncdc.gov.vn/viet-nam-full.html?startTime=2021-04-27&endTime=2022-03-07&provinces=-%2C29%2C&districts=-%2C%2C&tabKey=0
4.	Jakarta	Indonesia (Asia)	Major Outbreak	10.6 million	https://corona.jakarta.go.id/en
5.	London	UK (Europe)	Major Outbreak	7.6 million	https://coronavirus.data.gov.uk/details/download
6.	Manila City	Philippines (Asia)	Controlled outbreak	1.6 million	https://drive.google.com/drive/folders/1D0DuJQ_07tx9z-UdZG0OJqER0TKPWQ0F
7.	New Delhi	India (Asia)	Major Outbreak	10.9 million	https://prsindia.org/covid-19/cases
8.	New York City	USA (America)	Major Outbreak	8.2 million	https://catalog.data.gov/dataset/covid-19-daily-counts-of-cases-hospitalizations-and-deaths
9.	Singapore	Singapore (Asia)	Controlled Outbreak	5.9 million	https://ourworldindata.org
10.	Tokyo	Japan (Asia)	Major Outbreak	8.3 million	https://www.mhlw.go.jp/stf/covid-19/open-data.html

a Estimated rounded-off population statistics were collected from Worldometer (https://www.worldometers.info/) and/or official government sources.

### Parameters assessed

A range of parameters were assessed for each city/province to obtain a comprehensive overall picture of the measures and their impact on the transmission and spread of COVID-19 in the cities/provinces. The following parameters were assessed:

1. *Nature and duration of lockdowns*: Social distancing and isolation are the key pillars for transmission control of any airborne agent.^25^ A hard lockdown was defined as an all-stay-at-home restriction with only essential businesses open whilst a mild lockdown was defined as a lockdown period where work and travel were permitted with valid reasons. Since the nature of “valid reasons” remained unique to each city/province, we considered allowing dine-in at restaurants, visiting shopping malls, beauty parlors, massage parlors, barbershops, religious places, sports venues, gymnasiums, travelling to/from school and after-school activities under sanitary conditions (distancing, use of hand sanitizers, etc.) within the scope of mild lockdowns.
2. *Restriction implementation and monitoring:* For any policy to be effective, robust monitoring facilities and policies must be implemented. Hence, we investigated how the home-isolated patients were monitored with the help of police or doctors, hotline services to report violations of lockdowns, as well as the establishment of a COVID-19 task force that recommended and enforced regulations based on evolving epidemiological situation.
3. *Mass testing strategy*: Tracing and isolating high-risk individuals is necessary to stop the further spread of the virus.^25^^,^^26^ We analyzed which types of tests were being used majorly in the cities/provinces, along with the provision of drive-thru testing to fasten and ease testing. Drive-thru testing could potentially serve as a safer and much more efficient testing measure.^27^ We also investigated how often people in essential businesses were tested.
4. *Management of symptomatic patients:* We analyzed whether all symptomatic patients were tested and whether those with symptoms, but negative COVID-19 tests were isolated. Given the high false-negative rates for testing,^28^ isolation of symptomatic patients with negative tests may prove to be an important control measure during a major outbreak. Additionally, we looked at the location where the person was isolated (hospital/home, etc.) and whether family members could care for the patient or not.
5. *Tracing and isolation of close contacts:* Close contacts of confirmed cases especially within the first few days of symptom onset are at high risk of acquiring the virus,^29^ and hence, appropriate tracing becomes necessary. We investigated the tracing policies before and during the outbreak, along with the isolation policies for close contacts.
6. *Cluster identification and mitigation*: Indoor settings and areas with improper ventilation, overcrowding, high population turnover, and frequent movements are breeding grounds for the virus.^30^ Hence, we gathered reports of the authorities’ describing clusters in schools, prisons, slums, etc., and the mitigation steps that were taken by the authorities.
7. *Sufficient healthcare resources*: Overstraining of hospitals and staff are associated with excessive deaths.^31^ Hence, to assess this parameter, we gathered reports of whether patients were being treated or gathered in corridors or without beds. Reports of shortage of oxygen, essential medicines, etc., were also considered indicative of this parameter.
8. *Vaccination promotion*: Vaccination provides an effective and proven prophylaxis in the fight against COVID-19. However, misinformation and public mistrust led to the development of vaccine hesitancy. This coupled with already stretched healthcare resources would lead to multi-tier challenges in achieving herd immunity. We, hence, investigated the vaccination rates in the cities/provinces.

### Search strategy, data extraction, and validation

A team of 5-10 investigators was assigned to a single city/region. Each team was tasked with the search and collection of the answers to the questions using only publicly and freely available information sources including official government sources (Department of Health/Ministry of Health portals/websites/Twitter/Facebook or any other official social media accounts), official COVID-19 taskforces (if any), and leading national and international daily newspapers and media sources. All information collected was validated by the local team collaborators from the respective countries/cities/provinces.

### Role of the funding source

The present study did not receive any external funding.

## Results

**Figure 1:**
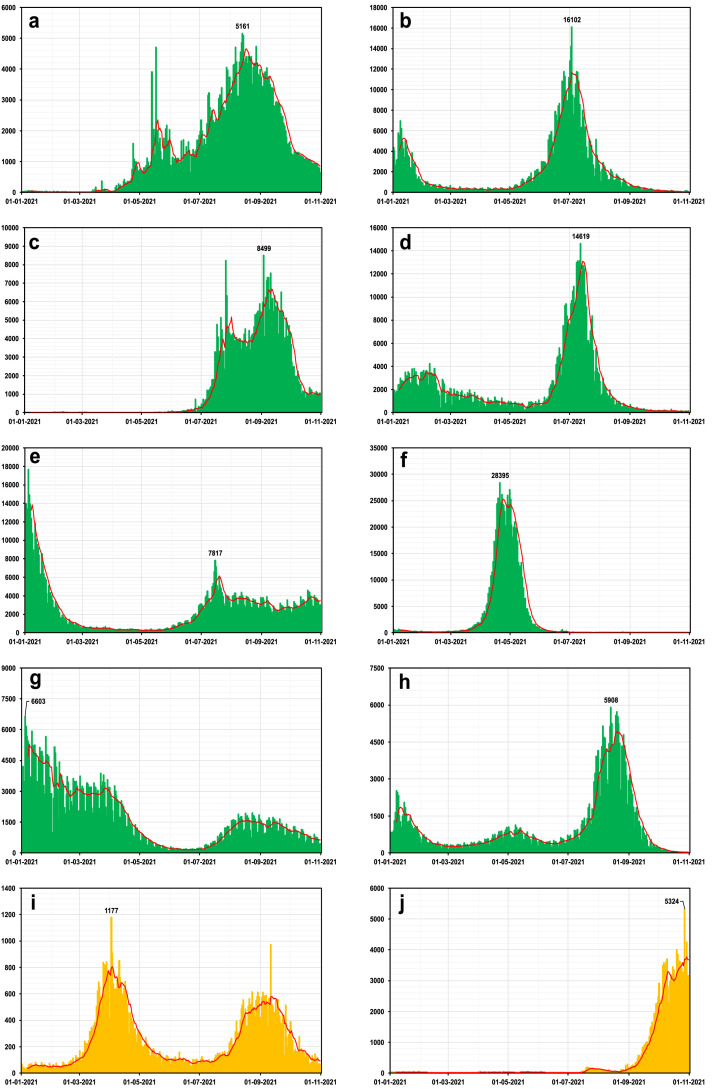
Trend graph showing the daily confirmed COVID-19 cases from January 2021 to October 2021 for cities/provinces with major outbreaks (green) and controlled outbreaks (yellow) with the red line indicating 7-day moving average. The peak highest daily number of confirmed cases is highlighted in the graph. (**a**) Bangkok; (**b**) Gauteng; (**c**) Ho Chi Minh City; (**d**) Jakarta; (**e**) London; (**f**) New Delhi; (**g**) New York City; (**h**) Tokyo; (**i**) Manila City and (**j**) Singapore. Note that the X-axis shows the date in DD.MM.YYYY format whilst the Y-axis shows the number of confirmed daily positive cases.

Our results indicate that most of the investigated cities/provinces with major outbreaks suffered from extended durations of the outbreaks that were sustained for more than 100 days (out of a total of 304 days between Jan 2021 and Oct 2021). New Delhi and Tokyo were the only cities that had smaller durations of outbreaks despite having major outbreaks (66 and 86 days, respectively). Both control cities, Singapore and Manila City, suffered from relatively shorter durations of the outbreak (Table 2 and Figure 1). Although New Delhi reported the highest total number of confirmed cases in the investigated period (814,397 confirmed cases), upon adjustment for population, it was Ho Chi Minh City with the highest number of confirmed cases per 100,000 population (12,291.03 cases per 100,000 population). Though Singapore reported more than 5000 confirmed cases/day, due to its peak towards the ambiguous Delta/Omicron border, we still considered it as a city with a controlled outbreak for this study.

**Table 2:** COVID-19 outbreak characteristics during the investigated period for the cities/provinces included in the study.

City/Provinces	Duration of the outbreak (in days)^a^	Peak daily cases reported (on date)	Total number of confirmed cases^b^	Confirmed cases per 100,000 population
Bangkok	155	5161 (13th Aug)	426,155	7748.27
Gauteng	132	16,102 (3rd July)	632,925	4083.39
Ho Chi Minh City	107	8499 (3rd Sept)	430,186	12,291.03
Jakarta	160	14,619 (11th July)	677,805	6394.39
London	190	7817 (15th July)	788,348	10,373.00
Manila City	01	1177 (2nd April)	75,978	4748.63
New Delhi	66	28,395 (20th April)	814,397	7471.53
New York City	181	6603 (4th Jan)	518,811	6326.96
Singapore	43	5324 (27th Oct)	139,775	2369.07
Tokyo	86	5908 (13th Aug)	320,407	3860.33

a Duration of outbreak is defined as the number of days with equal to or greater than 1000 daily confirmed positive COVID-19 cases between 1st January 2021 and 31st October 2021.

b Total number of confirmed cases is sum of total daily confirmed positive COVID-19 cases between 1st January 2021 and 31st October 2021.

### The Swiss cheese model for COVID-19 transmission

**Figure 2:**
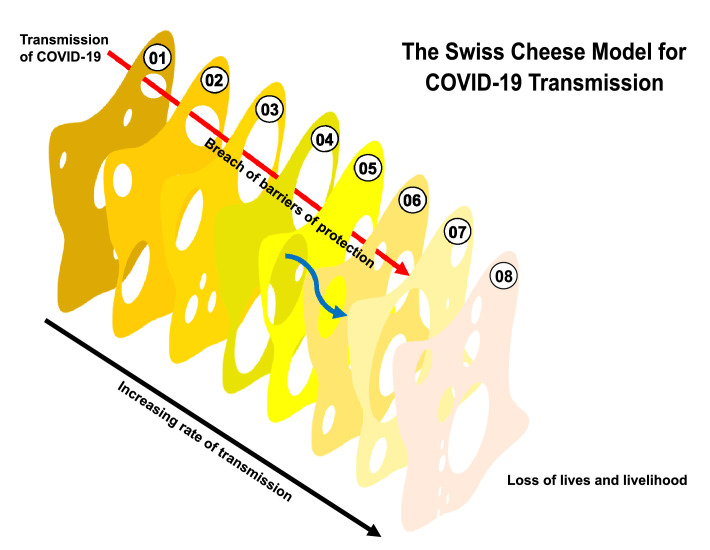
The Population-level Swiss Cheese Model for COVID-19 transmission. Each cheese slice indicates specific barriers for protection and prevention with holes representing breaches (or failures) in the implementation of the specific barrier - (**1**) Planning, swiftly acting, and adapting to outbreaks; (**2**) Rapid enforcement of public health measures (like mask wearing, social distancing); (**3**) Sufficient testing capacity; (**4**) Proper isolation of the confirmed patients; (**5**) Robust identification and isolation measures for close contacts (contact tracing); (**6**) Cluster identification and mitigation to prevent high co-worker and household transmission rates; (**7**) Sufficient healthcare resources (like beds, oxygen, respirators); and (**8**) Vaccination promotion. Some of the barriers are co-enforceable like contact tracing and cluster identification (blue arrow). With widespread breaches in multiple barriers, the risk of viral transmission increases manyfold (black arrow), which allows for easy spread of the virus amongst the community (red arrow); ultimately leading to loss of lives and livelihood.

First proposed by James Reason,^32^ the Swiss cheese model is an excellent demonstrator for illustrating the differences in the COVID-19 transmission rates and daily confirmed cases amongst the investigated cities/provinces.^33^^,^^34^ Each layer of the cheese represents a barrier (or measure) for protection and prevention (Figure 2), thereby highlighting that the prevention of COVID-19 transmission relies on a multi-tier systemic and collective response system from all sections of the society. The impact of failures (as represented by holes) in one intervention/layer would be minimized by the next intervention only if the holes do not overlap each other, in which case, there would be a higher likelihood of Covid-19 transmission. The staggering increase in daily case counts in different cities/provinces is often the result of a combination of failures in different interventions and seldom due to a single enforcement cause.

### Nature and duration of lockdowns

Travel and mobility restrictions have been long proposed and implemented in the management of airborne infectious agents including SARS, influenza, etc.^35^^,^^36^ Such restrictions reduce the chances of any individual leaving an outbreak area, thereby reducing the incidence of imported cases in a non-source region.^35^ Nonetheless, proper planning, swift action, and adaptation (first layer of the Swiss cheese model; Figure 2) are crucial for successfully implementing such restrictions. It has been demonstrated that only mobility restrictions of the magnitude >99% are truly effective in increasing the time between the export of the cases to the order of weeks.^35^^,^^36^ A lockdown of this magnitude would correspond to the hardest form of restrictions whereby only essential services (hospitals, police, grocery stores, etc.) are allowed to work in an epidemiologically safe environment. Such strict restrictions were implemented in five of the ten investigated cities/provinces. In Bangkok and Singapore, the duration of the hard lockdown was the longest with respect to number of outbreak days (79% and 70% of total outbreak days, respectively), followed by New Delhi and Ho Chi Minh City at 65% and 64% of total outbreak days, respectively (Table 3). Gauteng had imposed a hard lockdown only for about a third of the number of outbreak days. Jakarta, Tokyo, London, Manila City, and New York City did not impose hard lockdowns but mostly followed imposing mild lockdown strategy. In New Delhi, on the other hand, a locality-wise lockdown strategy was implemented whereby micro-cluster areas were under hard lockdown whilst the rest of the city was under mild lockdown. Manila City implemented a bubble called NCR+ (National Capital Region) that allowed most businesses in the bubble areas to remain operational but imposed strict movement controls in and out of the bubble.

**Table 3:** Distribution of nature and duration of lockdowns.

Cities/Provinces	Hard Lockdown^a^		Mild Lockdown^a^	
	Duration (in days)	% Days of outbreak	Duration (in days)	% Days of outbreak
Bangkok	123	79%	32	21%
Gauteng	28	31%	62	69%
Ho Chi Minh City	69	64%	39	36%
Jakarta	0	0%	160	100%
London	0	0%	190	100%
Manila City	0	0%	Followed a bubble strategy called NCR+	
New Delhi	43	65%	Follows locality-wise lockdown strategy	
New York City	0	0%	181	100%
Singapore	30	70%	13	30%
Tokyo	0	0%	86	100%

a Hard lockdowns were defined as an all stay at home restriction with only essential business open whilst a mild lockdown was defined whereby work and travel were permitted with valid reasons.

### Restriction implementation and monitoring

Despite achieving complete hard lockdowns, travel restrictions and social isolation can only slow down the transmission rather than halting it altogether.^35^ Public compliance and monitoring of the lockdown violations are equally necessary for achieving the desired results (second layer of the Swiss cheese model; Figure 2). However, enforcement of home isolation for millions of residents poses a huge logistical and manpower challenge. There are multiple ways of enforcing restrictions, some of which include police patrolling, hotlines for reporting violations, and the establishment of an independent COVID-19 task force, which are under the jurisdictions of individual cities/provinces (Table 4).

**Table 4:** Measures implemented for restriction monitoring.

Cities/Provinces	Police patrolling to ensure containment	Hotlines/Apps for violation reporting	Independent COVID-19 task force
Bangkok	Yes, only during night curfew daily	Yes	Yes
Gauteng	Yes	Yes	Yes
Ho Chi Minh City	Yes, only during hard lockdown	No	No
Jakarta	Yes, closure of multiple main streets	Yes	Yes
London	No patrolling	Yes	Yes
Manila City	Yes, door-to-door suspected COVID-19 case search	Yes	No
New Delhi	Yes, maintaining containment areas	Yes	Yes
New York City	No patrolling	Yes	No
Singapore	No patrolling	Yes	Yes
Tokyo	No patrolling	No	Yes

Four cities (London, Singapore, New York City, and Tokyo) opted-out in terms of implementing police patrolling the streets and homes. Health conditions of home-isolated patients were checked by phone, SMS, and applications in Tokyo and Singapore. Other cities meanwhile implemented patrolling in a more personalized manner. Bangkok used patrolling only during nights, whilst Ho Chi Minh City used patrolling only during hard lockdowns to place seals on the doors of the confirmed cases. Gauteng and Jakarta meanwhile deployed also armed forces to bolster the patrolling. New Delhi used a mix-model approach where it used police to isolate communities, but local medical officers visited the house of positive individuals to stamp their hands and paste isolation certificates outside the residence. This strategy of patient monitoring by healthcare officials was also echoed in Bangkok via telecommunications and video calls.

### Mass testing strategy

Testing the public-at-large is a potent strategy to reset the pandemic since it allows cities/provinces to identify hotspots and appropriately begin the process of contact tracing to break the chains of viral transmission and restart economic activities.^37^ However, if not delivered to the right people at the right time, simply finding more cases could prove to be counter-productive and make things worse.^37^^,^^38^ Furthermore, the differences in the specificity and sensitivity of the different types of available tests (lateral flow, rapid antigen test [RAT], real-time polymerase chain reaction [RT-PCR], etc.) make it difficult to detect infectious people (high viral loads) from ones that are not so infectious (low viral loads). Hence, having appropriate and sufficient testing capacity is vital for successful screening and isolation of the population (third layer of the Swiss cheese model; Figure 2). All cities/provinces except Tokyo (Table 5), relied on using mass testing strategies comprising both rapid antigen tests and standard RT-PCRs. Majorly RATs were preferred and mass-used due to their low cost and fast results turnaround. PCR was being employed for either select cases (like to confirm results of RATs) or in cases where there was high suspicion. In London, for example, RATs were made freely available for people for self-testing irrespective of their symptoms, whilst PCR was employed for confirmation of positive RAT. In New Delhi, on the other hand, RATs were employed primarily for non-containment localities whilst PCR was employed for containment localities and/or confirmation of positive RAT.

**Table 5:** Descriptive findings regarding the mass testing and contact tracing strategy in individual cities/provinces between January and October 2021.

Cities/Provinces	No. of laboratory tests conducted	Average No. of daily tests conducted	No. of tests per 1000 population	No. of total laboratory positive cases	Cases per 1000 population	Total test positivity rate^b^^,^^c^
Bangkok^a^	-	-	-	426155	77.48	-
Gauteng	3715555	12222	239.71	632925	40.83	17.03%
Ho Chi Minh City^a^	-	-	-	430186	122.91	-
Jakarta	6065249	19952	572.19	677805	63.94	11.12%
London	19028110	62593	2503.70	788348	103.73	04.14%
Manila City	2317228	7622	1448.27	75978	47.49	03.28%
New Delhi	20619994	67828	1891.74	814397	74.72	03.95%
New York City	23787885	78250	2900.96	518811	63.27	02.18%
Singapore	15398343	50652	2609.89	139775	23.69	00.91%
Tokyo	2948400	9699	355.23	320407	38.60	10.87%

a For Bangkok and Ho Chi Minh City, only national country-wide statistics for number of tests conducted were available.

b WHO had previously suggested a positivity rate of around 3–12% as a general benchmark of adequate testing, along with recommending that test positivity should remain at 5% or lower for 14 days before regions reopen (Source: https://globalhealth.harvard.edu/evidence-roundup-why-positive-test-rates-need-to-fall-below-3/).

c The total test positivity rate is calculated for the period between January and October 2021 and gives the overall positivity rate. It doesn't represent the individual fluctuations in specific weeks during these months.

Since essential businesses were opened throughout the different phases of the pandemic (irrespective of the nature of lockdowns), they represent one of the most epidemiologically at-risk populations and a priority group in mass-testing strategies. In all cities/provinces except Ho Chi Minh City, people in essential businesses were only tested when symptomatic and/or when they were in close contact with a laboratory-confirmed positive case, though all cities/provinces encouraged self-testing. Ho Chi Minh City recommended testing of people in essential businesses every 2–5 days with RATs and occasionally with PCR. Finally, simply having a policy in place is not enough, it is equally important to get the tests to the masses. Apart from the universal availability of self-testing kits, all cities/provinces provided residents with more convenience by offering drive-thru testing facilities in hospitals, laboratories, and clinics.

### Management of symptomatic patients

The fourth layer of the Swiss cheese model relates to the management of symptomatic patients (Figure 2). In all cities/provinces except Singapore, testing was done for all symptomatic patients (having fever, cough, sore throat, changes in smell, taste, etc.) using RAT and/or PCR. Singapore tested symptomatic people voluntarily along with providing patients with respiratory symptoms a medical certificate that was valid for five days. In case the symptoms did not improve after 5 days, the patient would be referred for further assessment. In Bangkok and London, RATs were compulsory if patients were symptomatic (followed by PCR confirmation for positive RATs) or were admitted to the hospital. For elective procedures like endoscopy, PCR testing was also done a few days before the procedure whilst testing was voluntary for people with negative RATs but presenting with symptoms.

Symptomatic patients with a negative COVID-19 test were mandated to be isolated at home in most of the cities, however, Singapore, Tokyo, Gauteng, and Jakarta only issued advisory to voluntarily isolate at home. In Jakarta retesting of RATs was advised to be undertaken 2-3 days after the first testing for the symptomatic patients (in case of negative cases) and isolated in case of a further positive test. For confirmed infected patients, isolation was mandatory at home, hotels, hospitals, and/or designated field facilities in all the cities/provinces. Close contacts, only if from the same household or same family, were allowed to be isolated with the primary infected person. Relatives were not allowed to take care of the COVID-19 patients in the hospitals except in New Delhi, New York City, and Jakarta. In New Delhi, only mothers were allowed inside the wards in case of children without the need of prior approval whilst all other requests were granted on a case-by-case basis. Jakarta allowed only one relative to stay with the patient (no specific criteria) and he/she must stay until the end of the treatment of the patient (caretaker rotations were not allowed).

### Tracing and isolation of close contacts

It is crucial to promptly identify and manage the close contacts of confirmed cases to support early diagnosis and interrupt the onward transmission chain.^39^ It requires a high level of public awareness and easy access to testing as discussed above. Hence, it represents the fifth layer of our Swiss cheese model (Figure 2). Before the start of the delta waves, all cities/provinces except Jakarta had quite intensive levels of contact tracing. Volunteers were roped in Bangkok, Jakarta, and Gauteng to facilitate effective contact tracing, whilst New Delhi and Tokyo had an expanded testing and scanning strategy at entry and exit points of places of major public gatherings. New York City employed a “Testing, Tracing & Take Care” strategy on similar lines to other cities. In addition to the use of the application, mainly oriented as the contact tracing and check-point monitoring tool, Jakarta also used phone and internet contact tracing for the relatives of COVID-19 positive patients. Although the mass testing rate in Jakarta was higher than WHO's standards (Table 5), it failed to meet the benchmark of tracing up to 30 close contacts per confirmed case, indicating a weak level of contact tracing.^40^^,^^41^

During the wave peaks, Ho Chi Minh City, Bangkok, Jakarta, and New Delhi failed to keep up with the contact tracing. All these cities gave up a lot on contact tracing. In some instances, even the symptomatic patients could not get tested. In Manila City, F1 contact tracing teams were notifying the contacts of confirmed cases. Singapore, Tokyo, and Gauteng meanwhile intensified their contact tracing during the wave peaks. London and New York City maintained the same intensity of contact tracing as before the wave peaks. In all cities/provinces, close contacts were isolated at home and/or designated facilities. However, in New Delhi, home isolation was instructed only in cases of asymptomatic and mild cases and in the presence of a caregiver. Close contacts from the same household (family members) were allowed to isolate with the primary infected person in all cities/provinces. Though, in such a case, the individuals should isolate in separate individual rooms. In all other cases, the close contacts were not allowed to isolate with the infected person, except in Ho Chi Minh City and Tokyo where sometimes close contacts were isolated with suspected cases.

### Cluster identification and mitigation

A case cluster could be defined as a group of ≥ 5 confirmed cases that shared a common transmission route but excluded cases with secondary epidemiological links such as within-household transmission.^42^^,^^43^ Furthermore, clusters in nursing homes or healthcare facilities inheritably have different transmission characteristics in terms of population susceptibility and infection nodes available,^44^ which takes them out of the equation. Our focus was on community outbreaks and clusters that arose in cramped, highly frequented, and poorly ventilated high-risk zones (sixth layer of Swiss cheese model; Figure 2). In all cities/provinces, large clusters of COVID-19 cases were identified in high-risk zones like slums, and prisons. The situation in Bangkok, New Delhi, and Singapore was of extreme concern with some estimates forecasting a 40–50% positivity rate in such regions. To mitigate and prevent a further surge in high-risk zones, several strategies were employed by the local authorities. Whilst Ho Chi Minh City and London resorted to only testing for cases, Gauteng imposed a Draconian lockdown in those zones. Jakarta implemented the Community Activities Restriction Enforcement (CARE) in January 2021 which was further strengthened in July 2021 to tackle large clusters. Priority vaccination, targeted aggressive testing, and recommendation of self-isolation of people from those zones was done in Manila, Tokyo, Jakarta, and Bangkok. Singapore restricted and suspended visits to residential care homes along with reporting temperature and oximeter readings twice daily from suspected patients. New Delhi and New York City took additional steps to decongest the prisons by releasing inmates on emergency bail and/or parole.

### Sufficient healthcare resources

Clinically, confirmed cases are the priority for any treatment protocol since prompt antiviral treatment administration reduces clinical severity and infectiousness.^45^ However, a rapid mass influx of patients and lack of healthcare resources impede this goal. The lack of proper equipment, workers, drugs, ICU beds, ventilators, etc., highlights the critical need for investment and upgradation of the healthcare facilities in all the cities/provinces. Hospitals, clinics, and other health institutions remained overstretched and overburdened during the wave period with multiple reports from all cities/provinces recording poor bed-to-patient ratios, leading to longer waiting, refusal of treatment as well as gathering of patients in the corridors. The situation in New Delhi was extremely severe with multiple patients dying outside the hospitals due to a shortage of healthcare providers and beds. A similar scenario was witnessed in Jakarta with many patients unable to enter the hospitals or were searching for multiple hospitals just to wait outside. ICU rooms were overloaded, with many patients being treated in tents, and many simply lay in their homes without any specific monitoring and treatment. The oxygen supply was also very scarce, even in the hospitals. An overview of the healthcare resources available in the investigated cities/provinces is shown in Table 6.

**Table 6:** Healthcare resources available in the investigated cities/provinces.

Cities/Provinces	No. of Public Hospitals	No. of Private hospitals	Total No. of hospitals^a^	No. of ICU beds^a^	No. of hospital beds^a^	No. of doctors per 1000 inhabitants (country-level)
Bangkok	48	116	164	262	69682	0.81
Gauteng	39	83	122	1462	30934	0.91
Ho Chi Minh City	82	46	128	370	30000	0.82
Jakarta	49	144	193	921	23081	0.43
London	-	-	134	-	37878	2.81
Manila NCR	16	43	59	1264	9421	0.60
New Delhi	37	43	80	2222	9581	0.86
New York City	-	-	70	2059	15338	2.61
Singapore	14	9	23	163	13614	2.29
Tokyo	-	-	650	1100	125700	2.41

a Note: The data presented doesn't include field hospitals, make-shift care centers, hotels, or other temporary facilities that were made available for use in the management of Covid-19 patients.

### Vaccination promotion

Vaccination against COVID-19 represents a key preventive measure to reduce disease burden, severity, and break transmission chains.^46^ Since the population-level immunity to COVID-19 is limited, vaccines can greatly relieve the hospital resources by reducing the incidence, hospitalizations, and deaths, especially amongst vulnerable hard-to-manage populations (eighth layer of the Swiss cheese model; Figure 2).^46^^,^^47^ However, challenges like inequitable vaccine distribution, limited healthcare workers to administer vaccines, misinformation on social media, and general vaccine hesitancy have impacted the mass rollout of vaccines. In all our cities/provinces, by October 31^st^, 2021, at least 70% of the population was partially vaccinated (dose 1) except in Gauteng and Manila NCR (Table 7). Bangkok, Jakarta, and Ho Chi Minh City managed to vaccinate more than 91% of their populations with one dose. In terms of a fully vaccinated population (dose 1+2 of Pfizer/Moderna/AstraZeneca/ Sinopharm/Sputnik V or dose 1 of Johnson & Johnson), most cities had managed to vaccinate 65% of their population. Gauteng and Manila NCR remained exceptions here as well, with Gauteng having exceptionally low vaccination cover (Table 7). Ho Chi Minh City, Singapore, Jakarta, London, and Tokyo all had managed to fully vaccinate more than 70% of their population.

**Table 7:** Population that was vaccinated against COVID-19 in the investigated cities.

Cities/Provinces	Date of start of mass vaccination^b^	Partially Vaccinated (Dose 1)^a^		Fully Vaccinated (Dose 1+2)^a^	
		No. of people	% City population	No. of people	% City population
Bangkok^c^	07.06.2021	13.61 million	91.62%	10.40 million	70.01%
Gauteng	17.05.2021	3.98 million	25.68%	2.07 million	13.35%
Ho Chi Minh City^e^	10.07.2021	-	> 95%	-	79.00%
Jakarta	08.06.2021	10.10 million	95.28%	7.80 million	74.28%
London	18.06.2021	6.05 million	79.60%	5.46 million	71.84%
Manila NCR^d^	11.10.2021	9.08 million	67.26%	7.90 million	58.52%
New Delhi^c^	01.05.2021	13.05 million	-	7.43 million	68.17%
New York City	06.04.2021	6.14 million	74.87%	5.50 million	67.07%
Singapore	03.02.2021	4.68 million	79.32%	4.49 million	76.10%
Tokyo^c^	21.06.2021	10.27 million	74.20%	9.85 million	71.20%

a Note that vaccination rates are approximate and include all approved COVID-19 vaccines and eligible age groups in the respective cities. The number of people represents the total number of people who were vaccinated from the respective date of start of vaccination to 31^st^ October 2021. Additionally, the data also includes non-residents of the city who were vaccinated in the city (reliable distribution is not available). Hence, % City population may not be precise, however, does provide an approximate estimation of the vaccination coverage in the city for inter-city comparisons.

b Mass vaccination refers to the period from which all adults aged 16+ or 18+ (based on different countries) were eligible for vaccination.

c For New Delhi, % City population vaccinated with first dose cannot be estimated due to number of doses administered surpasses total population. The vaccination coverage for Bangkok and Tokyo was obtained from official sources since the number of doses exceeded the total population of the city (https://ddc.moph.go.th/covid19-dashboard/?dashboard=province and https://stopcovid19.metro.tokyo.lg.jp/en/).

d For Manila, the statistics shown are for Manila NCR (National Capital Region, Metro Manila) with population of 13.5 million.

e For Ho Chi Minh City, only percentages are available from the official sources (https://luatvietnam.vn/y-te/bao-cao-1730-bc-byt-2021-tinh-hinh-dich-va-cong-tac-chong-tac-phong-chong-covid-19-ngay-30-10-2021-211811-d6.html).

United Kingdom (London) was the first country to approve Covid-19 vaccines in December 2020 (Table 8) followed closely by New York City and Singapore (Singapore was first in the Indo-pacific region). New York City, Tokyo, and Singapore approved only three vaccines each during the entire period of study. On the other hand, Jakarta had approved 11 vaccines during the same time (Table 8). All cities/provinces had approved a cocktail of different types of vaccines based on local regulations. Since the issuance of emergency use authorization (EUA) did not necessarily correlate with the availability of vaccines, it would be difficult to estimate the role of more vs fewer vaccines in the market. Generally, the mRNA-based vaccines (Comirnaty and Spikevax) were approved for use in 9 of the 10 investigated cities/provinces, whilst Vaxzervria and Janssen vaccines were approved for use in 8 of the 10 investigated cities/provinces.

**Table 8:** Covid-19 vaccines approved (emergency use authorization) in the investigated cities and provinces (date of approval# in dd/mm/yy format).

Cities/Provinces	mRNA based		Non-replicating viral vector			Inactivated		
	Pfizer/BioNTech Comirnaty	Moderna Spikevax	AstraZeneca/Oxford Vaxzervria^b^	J&J Janssen	Gamaleya Sputnik V^c^	Sinopharm Covilo	SinoVac CoronaVac	Bharat Biotech Covaxin
Bangkok	24.06.21	13.05.21	20.01.21	25.03.21	Pending^a^	28.05.21	22.02.21	Pending^a^
Gauteng	16.03.21	Pending^a^	23.01.21	22.02.21	Unknown^a^	07.02.21	03.07.21	Unknown^a^
Ho Chi Minh City‡	12.06.21	29.06.21	29.01.21	15.07.21	23.03.21	04.06.21	Unknown^a^	10.11.21
Jakarta$	15.07.21	02.07.21	09.03.21	07.09.21	24.08.21	30.04.21	11.01.21	Unknown^a^
London	02.12.20	08.01.21	30.12.20	28.05.21	Unknown^a^	Unknown^a^	Unknown^a^	Unknown^a^
Manila NCR‡	11.03.21	05.05.21	28.01.21	20.04.21	19.03.21	08.06.21	07.04.21	20.04.21
New Delhi†	Unknown^a^	29.06.21	03.01.21	07.08.21	13.04.21	Unknown^a^	Unknown^a^	03.01.21
New York City	11.12.20	19.12.20	Pending^a^	27.02.21	Unknown^a^	Unknown^a^	Unknown^a^	Pending^a^
Singapore	14.12.20	03.02.21	Unknown^a^	Unknown^a^	Unknown^a^	Unknown^a^	23.10.21	Unknown^a^
Tokyo	15.02.21	21.05.21	21.05.21	Unknown^a^	Unknown^a^	Unknown^a^	Unknown^a^	Unknown^a^

# Note that date of approval of vaccine doesn't indicate that the vaccine was made available for use for public. Additionally, date of approval doesn't correspond to the date of start of administration of vaccine. The date of approval is representative for the entire country and for adult populations (Source: https://covid19.trackvaccines.org/). The date of approval may vary by 1-2 days from official government sources (due to variations in news reporting and official dates).

a Pending status indicates that the vaccine had not approved been approved in the period studied in the present study (up to November 2021). Unknown status indicates no information is available regarding the application status and approval status for emergency use authorization in the country.

b AstraZeneca/Oxford Vaxzevria also includes its analogue produced by Serum Institute of India Covishield.

c Gamaleya Sputnik V doesn't include the later developed Sputnik Light.

‡ Vietnam (Ho Chi Minh City) had also approved Center for Genetic Engineering and Biotechnology (CIGB) Abdala on 18.09.21.

$ Indonesia (Jakarta) had also approved Anhui Zhifei Longcom Zifivax on 07.10.21, Serum Institute of India COVOVAX on 01.11.2021, CanSino Convidecia on 07.09.21 and BioKangtai KconecaVac on 31.10.21.

† India (New Delhi) had also approved Zydus Cadila ZyCoV-D on 20.08.21 and Sputnik Light on 17.05.21. ^‡^Philippines (Manila NCR) had also approved Sputnik Light on 23.08.21 and Serum Institute of India COVOVAX on 17.11.21.

## Discussion

The present study demonstrates the differences in the containment approaches and the resourcefulness of the Swiss Cheese model in illustrating the variations in the internal and external limitations that may have impacted successful management and containment of Covid-19. The proposed population-level Swiss cheese model shows that it is not one barrier that is responsible for reducing the spread of the virus, rather multiple barriers and the interactions between those barriers are the driving force in determining the final output. Furthermore, it demonstrates that even though public health measures such as contact tracing and vaccine promotion proved to be more effective than others like mass testing, single prevention method is not effective at reducing the spread of the virus, and it takes a collaborative effect of different interventions discussed in our manuscript to effectively break the chain of transmission. During the pandemic in 2021 mostly inter-country travel was restricted whilst intra-country travel was permitted. Hence, how an individual city handles the pandemic also affects how the entire country handles the pandemic since the chain of transmission needs to be broken at the very grassroot level to achieve nationwide control.

Nonetheless, the scope of the cities and provinces included in the present study limits our ability to generalize the findings and draw conclusive arguments in ascertaining the best or rather the most suitable strategies for reducing the spread of COVID-19 in communities. Several reasons can be attributed to this limitation. Firstly, though country-wise data is far more prominently available in governmental and media sources, the data extraction for city-level guidelines proved to be more challenging. However, analyzing city-level data in our opinion provides more situational awareness due to differences in communities, population densities, public opinion, healthcare facilities, lifestyle, environmental conditions, etc. The point here being cities/provinces represent a rather homogenous sample to study than an entire country. Secondly, some of the data were not readily accessible due to language barriers, non-publicly published information, etc. Thirdly, we investigated mostly cities/provinces with >5000 cases/day therefore, these cities/provinces have large populations and geographical spread. Fourthly, we only analyzed one side of the story, whilst the other side of public compliance, perception, and economic consequences need to also be factored in to completely understand the course of strategy that each of these cities/provinces partook. Finally, the ambiguity in the causative variant of a particular wave is difficult to completely capture.

Additionally, it's difficult to be extremely precise in comparing such approaches (given the nature of such types of studies) as the continuously evolving COVID-19 pandemic was and still is a very dynamic situation – and cities/provinces have continuously adapted to each new variant – in terms of both non-pharmaceutical (masking, social distancing, lockdowns) and pharmaceutical interventions (vaccines, antivirals, monoclonal antibodies). Equally important to highlight is the differences in the jurisdictional control of health measures in the investigated cities/provinces. Singapore for example, is a sovereign nation whilst New York and London are municipalities under both local and national restrictions. Similarly, New Delhi and wider National Capital Region (Delhi NCR) remains under the jurisdictional control of national, union territory, and regional restrictions. Such complexities naturally lead to two or more authorities to be at odds with each other, leading to a delay in the implementation of containment strategies.

Despite the limitations, previously reported ecological comparative review studies during the early stages of the Covid-19 pandemic^22^^,^^23^ have illustrated the utility and importance of studies like the present one. Our study adds to the Covid-19 literature by comparing less published East/Southeast Asian cities/provinces which controlled the virus much more effectively than their Western counterparts during the early pandemic, hence providing better insights into the management and control strategies.

Effective and timely measures are needed to control the transmission and spread of the COVID-19 virus. Whilst all the evaluated cities/provinces took a different personalized approach to managing the pandemic, certain measures like contact tracing, and vaccination promotion could prove to be more impactful to break the transmission chain than others like mass testing. Nonetheless, what remained common in all cities/provinces was the dynamic enforcement and monitoring of breaches of each barrier of protection. The measures taken to reinforce the barriers should be adjusted continuously based on the evolving epidemiological situation.

## Contributors

NJ and ICH contributed equally towards the manuscript. NTH conceptualized the study while NTH, NJ, and ICH were responsible for methodology. Data analysis was done by NJ and ICH. Data collection and curation was done by all authors. Validation of the study protocol was done by NTH whilst final validation of the results was done by TV, NJ, ICH, and NTH. Visualizations were done by NJ, and project supervision, investigations, and resource management were led by NTH. Original draft was prepared by NJ and ICH, whilst revisions and final editing were done by all authors. All authors have read and approved the final manuscript for publication.

## Data sharing statement

All data analyzed in the present study was gathered from media and government resources which are freely available in the public domain.

## Declaration of interests

The authors declare no conflicts of interests with regards to the present study. Authors did not receive any external funding for the study.
